# Abnormal Expression of DNA Double-Strand Breaks Related Genes, ATM and GammaH2AX, in Thyroid Carcinoma

**DOI:** 10.1155/2015/136810

**Published:** 2015-03-16

**Authors:** Jin-lin Hu, Si-si Hu, Xiu-xiu Hou, Xin Zhu, Jun Cao, Lie-hao Jiang, Ming-hua Ge

**Affiliations:** ^1^Department of Pathology, Zhejiang Province Cancer Hospital, Hangzhou 310022, China; ^2^Emergency Department, The First Affiliated Hospital of Wenzhou Medical University, Wenzhou 325000, China; ^3^Zhejiang Cancer Research Institute, Zhejiang Province Cancer Hospital, Hangzhou 310022, China; ^4^Department of Head and Neck Surgery, Zhejiang Province Cancer Hospital, Hangzhou 310022, China

## Abstract

ATM and *γ*H2AX play a vital role in the detection of DNA double-strand breaks (DSB) and DNA damage response (DDR). This study aims to investigate ATM and *γ*H2AX expression in thyroid cancer and discuss possible relationship between thyroid function tests and DNA damage. The expression of ATM and *γ*H2AX was detected by immunohistochemistry in 30 cases of benign nodular goiter, 110 cases of well differentiated thyroid cancer, 22 cases of poorly differentiated thyroid cancer, and 21 cases of anaplastic thyroid cancer. Clinicopathological features, including differentiation stages, distant metastasis, lymph node metastasis, T classification, TNM stage, and tests of thyroid functions (TPOAb, Tg Ab, T3, FT3, T4, FT4, TSH, and Tg), were reviewed and their associations with *γ*H2AX and ATM were analyzed. *γ*H2AX and ATM expressed higher in thyroid cancer tissues than in benign nodular goiter and normal adjacent tissues. *γ*H2AX was correlated with ATM in thyroid cancer. Both *γ*H2AX and ATM expression were associated with FT3. *γ*H2AX was also associated with T classification, TNM stage, FT4, TSH, and differentiation status. Therefore both of ATM and *γ*H2AX seem to correlate with thyroid hormones and *γ*H2AX plays a role in the differentiation status of thyroid cancer.

## 1. Introduction

Thyroid cancer, which is related to radiation exposure, represents the most common malignancy in the endocrine system. A marked increase in the incidence of thyroid cancer has occurred in recent years, in part due to the improvement in the diagnostic approach [1, 2]. Among them, anaplastic thyroid carcinoma is the most aggressive form and remains a medical challenge, with a poor survival time from diagnosis [3]. However, the genetic predisposition to thyroid cancer has still not been clearly elucidated.

DNA double-strand break (DSB), the primary lesions induced by radiation exposure, might be involved in thyroid carcinoma development. A single misrepaired DSB could be sufficient to lead to loss of large DNA fragments, abnormal mitosis, and even activation of cell death process [4, 5]. To reverse and diminish severe DNA damage, cells develop different mechanisms, including DNA recombination and repair, cell cycle arrest, and apoptosis [4]. Both ATM and H2AX have been proved to be implicated in maintaining genomic stability and response to DSB [6–8].

ATM gene deficiency leads to the syndrome named ataxia telangiectasia and the latter is characterized by genome instability, radiosensitivity, progressive ataxia, and susceptibility to malignancy [9]. After being recruited to the DSB sites, autoactivated ATM phosphorylates various substrates, including H2AX, NBS1, CHK2, and p53, thereby mediating DSB repairing [4, 8]. Some specific ATM alleles were correlated with the increased risk of various cancers, including lung, breast, and prostate cancer [10–12]. However, it has been proved that some specific ATM alleles could be protective in cutaneous melanoma [13]. Different studies demonstrated different roles of ATM in thyroid cancer. A research conducted by Wójcicka et al. revealed that the ATM variant ATM rs1801516 was not associated with PTC risk but could modify BRCA1 related risk [14]. Kang et al. also found no positive associations between ATM polymorphisms and PTC risk [15]. Some other researches demonstrated that ATM polymorphisms might be involved in the development of thyroid cancer [9, 16–18]. Accumulation of H2AX phosphorylation (*γ*H2AX), which could be mediated by ATM, has been detected in various cancers [19–22]. Activation of ATM and accumulation of *γ*H2AX could be a cellular response to DNA damage [6–8]. In the previous study, we have found that *γ*H2AX expression was increased in papillary thyroid cancer (PTC) patients and was reversely related to lymph node metastasis and TNM stage [23]. Recent researches demonstrated that *γ*H2AX was increased in human primary thyrocytes disposed by ionizing radiation and in differentiated thyroid cancer patients undergoing ^131^I therapy [24–26]. However, the role of ATM and *γ*H2AX in thyroid cancer has not been completely elucidated.

Various hormones, including estrogen, could induce DNA damage, thus contributing to carcinogenesis [27]. Recent studies found various thyroid disorders in thyroid cancer patients and found that abnormal FT3, FT4, and TSH levels were related to thyroid cancer risk [28]. The thyroid hormones exert momentous actions on energy metabolism. The increase in oxygen consumption led by thyroid hormones induces the generation of reactive oxygen species and reactive nitrogen species. And, in response, more cellular antioxidants could be consumed and antioxidant enzymes could be inactivated, thereby promoting oxidative stress and oxidative DNA damage. Thus, we hypothesize that there is a relation between thyroid disorders and DNA damage. However, there is lack of data about the relationship between thyroid functions and DSB-related genes.

To investigate the expression of *γ*H2AX and ATM in thyroid cancer, 30 cases of benign nodular goiter, 110 cases of well differentiated thyroid cancer, 22 cases of poorly differentiated thyroid cancer, and 21 cases of anaplastic thyroid cancer were included in this study. To determine if thyroid hormones play a role in DNA damage, data of thyroid function tests was collected and analysis was made to discuss the relationship between *γ*H2AX and ATM expression and thyroid function tests in thyroid cancer in this study.

## 2. Materials and Methods

### 2.1. Tissue Specimens

30 patients diagnosed with benign nodular goiter and 153 patients diagnosed with thyroid cancer were included in this study. Among the thyroid cancer group, 80 cases were diagnosed coexisting with benign nodular goiter. The thyroid cancer patients were identified between January 2001 and August 2014, while the benign nodular goiter patients were identified between January 2012 and June 2012 in Zhejiang Province Cancer Hospital, China. A final diagnosis of thyroid cancer and benign nodular goiter was confirmed by at least two experienced pathologists. Various clinicopathological features, including age, gender, differentiation stages, lymph node metastasis, and distant metastasis, were captured according to the medical records. T classification and TNM stage of the cancer patients were evaluated based on the 2002 edition of American Joint Committee on Cancer (AJCC) TNM staging criteria. Tests of thyroid functions, including peroxidase antibody (TPOAb), thyroglobulin antibody (TgAb), triiodothyronine (T3), basal plasma free T3 (FT3), thyroxine (T4), basal plasma free T4 (FT4), thyroid-stimulating hormone (TSH), and thyroglobulin (Tg) levels, were also studied prior to surgery and they were evaluated according to the following standards (Table 1). 30 patients diagnosed with benign nodular goiter, including 29 females and 1 male, ranged from 36 to 70 years old (49.0 ± 8.0). 153 patients diagnosed with thyroid cancer, including 104 females and 49 males, ranged from 18 to 78 years old (48.8 ± 13.9). Among the cancer patients, lymph node metastasis was found in 107 (69.9%) cases, while distant metastasis was detected in 19 (12.4%) cases. According to the postoperative histopathological examination, 110 (52.3%) cases were well differentiated, 22 (14.4%) were poorly differentiated, and the other 21 (13.7%) were anaplastic. None of all the thyroid cancer patients had ever received anticancer treatment before surgery.

### 2.2. Tissue Microarray Construction and Immunohistochemistry

By reviewing HE stained slides, paraffin blocks containing representative area from each specimens were chosen and extracted with the TM-1 tissue microarray kit (Changzhou Ruipin Precision Instrument Co., Ltd., China). The cores were subsequently placed and embedded in blank paraffin blocks. The blocks were incubated at 60°C for 30 minutes and cooled at room temperature for better fixation of the tissue.

3 *μ*m thick sections, obtained from tissue microarrays blocks or tissue paraffin blocks, were used for immunohistochemistry. The sections were then placed in xylene for deparaffinization and sequentially several graded alcohol dilutions for hydration. To detect *γ*H2AX expression, sections were boiled in a pressure kettle for 1.5 minutes in citrate buffer (pH 6.0) and cooled to room temperature for antigen retrieval. After endogenous peroxidase activity was blocked, the slices were incubated with anti-*γ*H2AX antibody (pS139, Cat:#2212-1, EPITOMICS, CA, USA) (1 : 200 dilution) for 90 minutes and subsequently the secondary antibody (DAKO, Denmark) for 30 minutes.

To detect ATM expression, sections were boiled in a microwave oven for 15 minutes in EDTA buffer (pH 9.0) for antigen retrieval and cooled to room temperature. After endogenous peroxidase activity was blocked, the slices were incubated with anti-ATM antibody (ab32420, Abcam) (1 : 200 dilution) for 90 minutes and subsequently the secondary antibody (DAKO, Denmark) for 30 minutes.

All slices were then treated with DAB for 5 minutes and subsequently counterstained with hematoxylin. All the sections were reviewed by at least two experienced pathologists. The positive cell percentages 0%, 0–25%, 26%–50%, 51−75%, and >75% were defined as 0, 1, 2, 3, and 4, respectively. The positive staining intensity were defined as 0, 1, 2, and 3 for no staining, light yellow, yellow brown and brown, respectively. Semiquantitative expression levels of ATM and *γ*H2AX were evaluated by multiplying the distribution and intensity score. Those with a final score <5 were defined as low expression and those ≧ 5 were considered high expression.

### 2.3. Statistical Analysis

All tests were two-sided and carried out with SPSS version 18.0. *γ*H2AX and ATM expression were compared between cancer tissues and benign tissues using *χ* ^2^ test. The correlation between *γ*H2AX and ATM was also evaluated by *χ* ^2^ test. In the cancer group, the associations between the expression of *γ*H2AX and ATM and clinicopathological features were carried out by *χ* ^2^ test. *P* ⩽ 0.05 was defined as statistically significant.

## 3. Results

### 3.1. Expression of *γ*H2AX and ATM in Thyroid Cancer and in Benign Tissues

**Figure 1:**
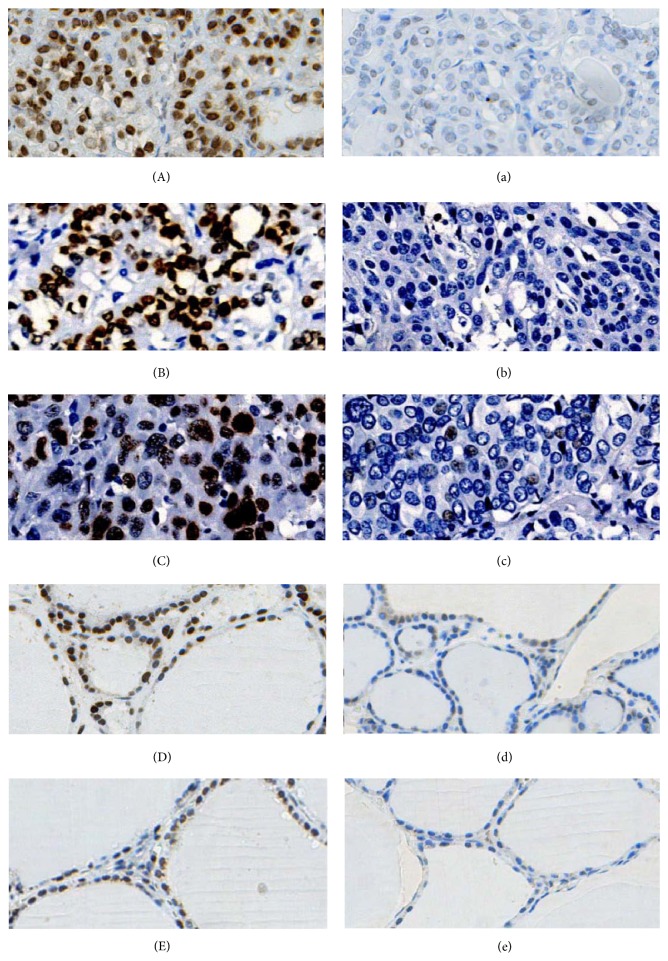
*γ*H2AX expression in thyroid tissues. (A) High expression of *γ*H2AX in well differentiated thyroid cancer. (a) Low expression of *γ*H2AX in well differentiated thyroid cancer. (B) High expression of *γ*H2AX in poorly differentiated thyroid cancer. (b) Low expression of *γ*H2AX in poorly differentiated thyroid cancer. (C) High expression of *γ*H2AX in anaplastic thyroid cancer. (c) Low expression of *γ*H2AX in anaplastic thyroid cancer. (D) High expression of *γ*H2AX in benign nodular goiter tissues. (d) Low expression of *γ*H2AX in benign nodular goiter tissues. (E) High expression of *γ*H2AX in normal adjacent tissues. (e) Low expression of *γ*H2AX in normal adjacent tissues. Original magnification of all images, ×200.

**Figure 2:**
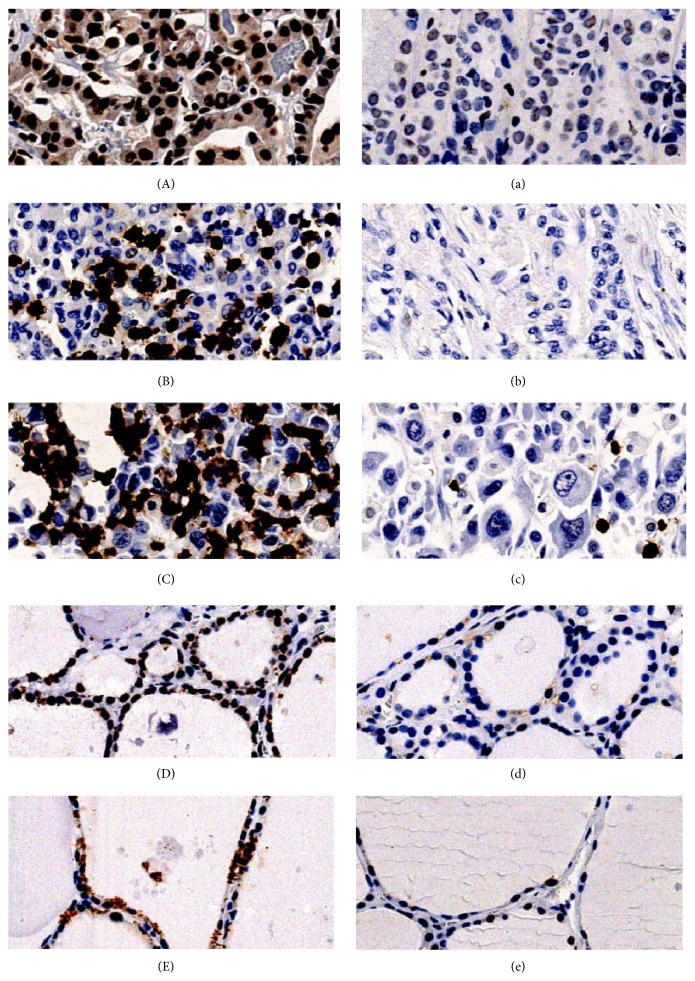
ATM expression in thyroid tissues. (A) High expression of ATM in well differentiated thyroid cancer. (a) Low expression of ATM in well differentiated thyroid cancer. (B) High expression of ATM in poorly differentiated thyroid cancer. (b) Low expression of ATM in poorly differentiated thyroid cancer. (C) High expression of ATM in anaplastic thyroid cancer. (c) Low expression of ATM in anaplastic thyroid cancer. (D) High expression of ATM in benign nodular goiter tissues. (d) Low expression of ATM in benign nodular goiter tissues. (E) High expression of ATM in normal adjacent tissues. (e) Low expression of ATM in normal adjacent tissues. Original magnification of all images, ×200.

*γ*H2AX was mainly expressed in nucleus (Figure 1), while ATM was mainly expressed in both nucleus and cytoplasm in thyroid tissues (Figure 2).

In benign nodular goiter patients, *γ*H2AX was highly expressed in 6 of 30 (20.0%) benign nodular goiter tissues and 3 of 30 (10.0%) normal adjacent tissues, with no statistical significance (*P* = 0.472), while ATM was highly expressed in 3 of 30 (10.0%) benign nodular goiter tissues and 2 of 30 (6.7%) normal adjacent tissues, with no statistical significance (*P* = 1.000).

In thyroid cancer, high *γ*H2AX expression was detected in 109 of 153 (71.2%) cancer tissues and 9 of 153 (5.9%) normal adjacent tissues, with statistical significance (*P* < 0.001), while high ATM expression was detected in 53 of 153 (34.6%) cancer tissues and 9 of 153 (5.9%) normal adjacent tissues, with statistical significance (*P* < 0.001).

Among 153 thyroid cancer patients, 80 cases were coexisting with benign nodular goiter. In this coexisting group, 76 of 80 (95.0%) cancer tissues and 10 of 80 (12.5%) benign nodular goiter tissues showed high expression of *γ*H2AX (*P* < 0.001). 31 of 80 (38.8%) cancer tissues and 3 of 80 (3.8%) benign nodular goiter tissues showed high expression of ATM (*P* < 0.001).

Overall, both *γ*H2AX and ATM expressed higher in thyroid cancer tissues than in benign nodular goiter and normal adjacent tissues, while benign nodular goiter and normal adjacent tissues showed no significant difference in *γ*H2AX expression. They showed no significant difference in ATM expression as well.

In thyroid cancer group, both ATM and *γ*H2AX were highly expressed in 45 cases and lowly expressed in 36 cases. Analysis between ATM and *γ*H2AX expression showed statistical significance (*P* = 0.007) (Table 2). ATM expression was correlated with *γ*H2AX expression in thyroid cancer in this study.

### 3.2. Clinical Significance of *γ*H2AX and ATM in Thyroid Cancer

Association between *γ*H2AX expression and various clinicopathological features of thyroid cancer was analyzed (Table 3). *γ*H2AX expression was related to T classification, and the level was higher in earlier T stage than that in later T stage (<0.001). *γ*H2AX expression was also correlated with TNM stage (<0.001) and differentiation status (<0.001). Well differentiated thyroid cancer tissues showed higher *γ*H2AX expression level than poorly differentiated thyroid cancer tissues, while the latter showed higher *γ*H2AX expression level than anaplastic thyroid cancer tissues. *γ*H2AX expression was also associated with several tests of thyroid functions including FT3 (*P* = 0.043), FT4 (*P* = 0.009), and TSH (<0.001). Other clinicopathological features showed no correlation with *γ*H2AX expression level, including gender, distant metastasis, lymph node metastasis, TPOAb, TgAb, T3, T4, and Tg.

Association between ATM expression and clinicopathological features of thyroid cancer was also analyzed (Table 4). Different pathologic type of thyroid cancer tissues showed different expression level of ATM, with statistically significant differences (*P* = 0.05). Anaplastic thyroid cancer tissues showed higher ATM expression than well differentiated thyroid cancer tissues, while the latter showed higher ATM expression than poorly differentiated thyroid cancer tissues. However no distinct correlation has been found between ATM expression and differentiation of thyroid cancer. ATM expression level was also associated with several tests of thyroid functions, including FT3 (*P* = 0.009) and T4 (*P* = 0.037). ATM expression level was not correlated with other clinicopathological features, including distant metastasis, T statues, lymph node metastasis, TNM stage, TPOAb, TgAb, T3, FT4, TSH, and Tg.

## 4. Discussion

ATM and *γ*H2AX are indicators of DSB and DDR. They are needed in the starting stage leading to DSB and could be early molecular events in carcinogenesis. H2AX is one of the substrates of ATM and could be phosphorylated by ATM. While autoactivated ATM could induce increased expression of *γ*H2AX, *γ*H2AX could in turn enhance the activity of ATM [29].

Abnormal expression of ATM and *γ*H2AX has been detected in various cancers with different roles. Some researches found that they were correlated with the increased risk of cancer [10–12, 19–22]. In this study, *γ*H2AX and ATM showed increased expression in thyroid cancer tissues as compared with benign tissues. And *γ*H2AX was correlated with ATM, which is in accordance with the previous study [29]. Both *γ*H2AX and ATM play a role in thyroid cancer.

*γ*H2AX was found to correlate with differentiation of thyroid cancer in this study. Well differentiated thyroid cancer tissues showed higher *γ*H2AX expression level than poorly differentiated thyroid cancer tissues, while the latter showed higher *γ*H2AX expression level than anaplastic thyroid cancer tissues. Statistically significant differences were also found in ATM expression among different pathologic types of thyroid cancer. Anaplastic thyroid cancer tissues showed higher ATM expression than well differentiated thyroid cancer tissues, while the latter showed higher ATM expression than poorly differentiated thyroid cancer tissues. Previous researches demonstrated that DSB-initiated genomic deletion, including ATM, was greatly increased in high-grade breast cancer [30]. Moreover, it has been proved that defects in ATM lead to poor differentiation of breast cancer [31]. Overexpression of *γ*H2AX has been proved to associate with differentiation and TNM stage in gastric cancer and could serve as a potential biomarker for gastric cancer [32]. In this study, *γ*H2AX and ATM expressed higher in well differentiated thyroid cancer tissues as compared to poorly differentiated thyroid cancer, which was consistent with the researches above. However, why anaplastic thyroid cancer tissue showed higher ATM expression than poorly differentiated thyroid cancer remains to be a problem. It is possibly because the specimens included in the study were insufficient. It is inappropriate to come up with a distinct correlation between ATM expression and differentiation of thyroid cancer. Anyway, more researches are needed to explore the potential relationship. Thus we proposed that *γ*H2AX was correlated with differentiation of thyroid cancer while ATM was not correlated with differentiation of thyroid cancer distinctly. *γ*H2AX was also found to be correlated with T classification and TNM stage. *γ*H2AX was increased in patients with earlier T stage. A previous study showed that *γ*H2AX was increased in premalignant lesions and *γ*H2AX might play a critical role in the early stage of cancer [33], which is in accordance with this study. We suggested that *γ*H2AX might play a more important role in early stage of thyroid cancer.

Thyroid hormones play a pivotal role in the metabolic activities and affect various organ systems. Zambrano et al. have proved that T3 could activate ATM-dependent adenosine monophosphate-activated protein kinase (PRKAA) signaling by combining with THRB (thyroid hormone receptor beta), thus playing a pivotal role in mitochondrial respiration and leading to DSB in mouse embryonic fibroblasts [34]. In the absence of ATM, T3 could not induce senescence, whereas transfection of ATM restored the response to T3 [34]. Associations between thyroid functions tests and ATM and *γ*H2AX expression were also analyzed in this study. We found no correlation between T3 and the expression of ATM and *γ*H2AX. However, the results showed that both of ATM and *γ*H2AX were correlated with FT3 in thyroid cancer. FT3 could be recognized as a marker of euthyroid syndrome in various diseases, and reduced FT3 in plasma could diminish the conversion of T4 to T3 [35]. Recent researches indicated that lower FT3 and FT4 levels and higher TSH level were correlated with thyroid cancer risk [28]. *γ*H2AX was also associated with FT4 and TSH in this study. Although the results are inconclusive, we speculated an association between thyroid disorders and ATM and *γ*H2AX expression.

Overall, both *γ*H2AX and ATM were supposed to influence thyroid hormones and *γ*H2AX has been proved to play a role in the differentiation status of thyroid cancer. Since ATM activation and *γ*H2AX have been recognized as indicators of DNA damage, more researches are needed for further discussing the relationship between thyroid functions and DNA damage.

## Figures and Tables

**Table 1:** The evaluation standards of the tests of thyroid functions.

Thyroid hormone	Level		Thyroid hormone	Level	
TgAb			TPOAb		
N	0–115	U/mL	N	0–34	U/mL
H	>115	U/mL	H	>34	U/mL
T3			T4		
L	<0.6	ng/mL	L	<4.5	*μ*g/dL
N	0.6–1.81	ng/mL	N	4.5–10.9	*μ*g/dL
H	>1.81	ng/mL	H	>10.9	*μ*g/dL
FT3			FT4		
L	<2.3	pg/mL	L	<0.89	ng/dL
N	2.3–4.2	pg/mL	N	0.89–1.76	ng/dL
H	>4.2	pg/mL	H	>1.76	ng/dL
TSH			Tg		
L	<0.35	*μ*IU/mL	L	<1.4	ng/mL
N	0.35–5.5	*μ*IU/mL	N	1.4–78	ng/mL
H	>5.5	*μ*IU/mL	H	>78	ng/mL

L represents low expression level; N represents normal expression level; H represents high expression level.

**Table 2:** Correlation between ATM and *γ*H2AX in thyroid cancer.

	Number	ATM-low		ATM-high		*P* value
		*N*	%	*N*	%	
*γ*H2AX-low	44	36	81.8%	8	22.2%	0.007
*γ*H2AX-high	109	64	58.7%	45	41.3%	

**Table 3:** Association between *γ*H2AX expression and clinicopathological features.

	Number	*γ*H2AX-low		*γ*H2AX-high		*P* value
		*N*	%	*N*	%	
Total number						
Distant metastasis						0.17
−	134	36	26.9%	98	73.1%	
+	19	8	42.1%	11	57.9%	
T classification						<0.001
1	11	0	0.0%	11	100.0%	
2	7	1	14.3%	6	85.7%	
3	84	13	15.5%	71	84.5%	
4	51	30	58.8%	21	41.2%	
Lymph node metastasis						0.1
−	46	9	19.6%	37	80.4%	
+	107	35	32.7%	72	67.3%	
TNM stage (2002 AJCC)						<0.001
I	49	5	10.2%	44	89.8%	
II	7	3	42.9%	4	57.1%	
III	42	5	11.9%	37	88.1%	
IV	55	31	56.4%	24	43.6%	
Differentiation						<0.001
Well differentiated	110	11	10.0%	99	90.0%	
Poorly differentiated	22	15	68.2%	7	31.8%	
Anaplastic	21	18	85.7%	3	14.3%	
TPOAb						0.376
N	120	30	25.0%	90	75.0%	
H	27	9	33.3%	18	66.7%	
TgAb						
N	119	30	25.2%	89	74.8%	0.455
H	28	9	32.1%	19	67.9%	
T3						
L	6	1	16.7%	5	83.3%	0.421
N	141	40	28.4%	101	71.6%	
H	2	0	0.0%	2	100.0%	
FT3						
L	10	5	50.0%	5	50.0%	0.043
N	130	31	23.8%	99	76.2%	
H	9	5	55.6%	4	44.4%	
T4						0.308
L	3	1	33.3%	2	66.7%	
N	133	34	25.6%	99	74.4%	
H	13	6	46.2%	7	53.8%	
FT4						0.009
L	7	4	57.1%	3	42.9%	
N	135	32	23.7%	103	76.3%	
H	7	5	71.4%	2	28.6%	
TSH						<0.001
L	7	6	85.7%	1	14.3%	
N	133	27	20.3%	106	79.7%	
H	9	8	88.9%	1	11.1%	
Tg						0.082
L	14	5	35.7%	9	64.3%	
N	97	20	20.6%	77	79.4%	
H	36	14	38.9%	22	61.1%	

**Table 4:** Association between ATM expression and clinicopathological features.

	Number	*γ*H2AX-low		*γ*H2AX-high		*P* value
		*N*	%	*N*	%	
Total number						
Distant metastasis						0.183
−	134	85	63.4%	49	36.6%	
+	19	15	78.9%	4	21.1%	
T classification						
1	11	9	81.8%	2	18.2%	0.287
2	7	6	85.7%	1	14.3%	
3	84	51	60.7%	33	39.3%	
4	51	34	66.7%	17	33.3%	
Lymph node metastasis						0.132
−	46	26	56.5%	20	43.5%	
+	107	74	69.2%	33	30.8%	
TNM stage (2002 AJCC)						0.602
I	49	33	67.3%	16	32.7%	
II	7	6	85.7%	1	14.3%	
III	42	26	61.9%	16	38.1%	
IV	55	35	63.6%	20	36.4%	
Differentiation						0.05
Well differentiated	110	70	63.6%	40	36.4%	
Poorly differentiated	22	19	86.4%	3	13.6%	
Anaplastic	21	11	52.4%	10	47.6%	
TPOAb						0.541
N	120	77	64.2%	43	35.8%	
H	27	19	70.4%	8	29.6%	
TgAb						0.231
N	119	75	63.0%	44	37.0%	
H	28	21	75.0%	7	25.0%	
T3						0.548
L	6	5	83.3%	1	16.7%	
N	141	91	64.5%	50	35.5%	
H	2	1	50.0%	1	50.0%	
FT3						0.009
L	10	8	80.0%	2	20.0%	
N	130	80	61.5%	50	38.5%	
H	9	9	100.0%	0	0.0%	
T4						0.037
L	3	3	100.0%	0	0.0%	
N	133	89	66.9%	44	33.1%	
H	13	5	38.5%	8	61.5%	
FT4						0.448
L	7	3	42.9%	4	57.1%	
N	135	89	65.9%	46	34.1%	
H	4	5	125.0%	2	50.0%	
TSH						0.448
L	7	6	85.7%	1	14.3%	
N	133	85	63.9%	48	36.1%	
H	9	6	66.7%	3	33.3%	
Tg						0.505
L	14	11	78.6%	3	21.4%	
N	97	61	62.9%	36	37.1%	
H	36	24	66.7%	12	33.3%	
